# Structural Determinants of the Outer Shell of β-Carboxysomes in *Synechococcus elongatus* PCC 7942: Roles for CcmK2, K3-K4, CcmO, and CcmL

**DOI:** 10.1371/journal.pone.0043871

**Published:** 2012-08-22

**Authors:** Benjamin D. Rae, Benedict M. Long, Murray R. Badger, G. Dean Price

**Affiliations:** Division of Plant Science, Research School of Biology, The Australian National University, Canberra, ACT 0200, Australia; Montana State University, United States of America

## Abstract

Cyanobacterial CO_2_-fixation is supported by a CO_2_-concentrating mechanism which improves photosynthesis by saturating the primary carboxylating enzyme, ribulose 1, 5-bisphosphate carboxylase/oxygenase (RuBisCO), with its preferred substrate CO_2_. The site of CO_2_-concentration is a protein bound micro-compartment called the carboxysome which contains most, if not all, of the cellular RuBisCO. The shell of β-type carboxysomes is thought to be composed of two functional layers, with the inner layer involved in RuBisCO scaffolding and bicarbonate dehydration, and the outer layer in selective permeability to dissolved solutes. Here, four genes (*ccmK2-4, ccmO*), whose products were predicted to function in the outer shell layer of β-carboxysomes from *Synechococcus elongatus* PCC 7942, were investigated by analysis of defined genetic mutants. Deletion of the *ccmK2* and *ccmO* genes resulted in severe high-CO_2_-requiring mutants with aberrant carboxysomes, whilst deletion of *ccmK3* or *ccmK4* resulted in cells with wild-type physiology and normal ultrastructure. However, a tandem deletion of *ccmK3-4* resulted in cells with wild-type carboxysome structure, but physiologically deficient at low CO_2_ conditions. These results revealed the minimum structural determinants of the outer shell of β-carboxysomes from this strain: CcmK2, CcmO and CcmL. An accessory set of proteins was required to refine the function of the pre-existing shell: CcmK3 and CcmK4. These data suggested a model for the facet structure of β-carboxysomes with CcmL forming the vertices, CcmK2 forming the bulk facet, and CcmO, a “zipper protein,” interfacing the edges of carboxysome facets.

## Introduction

Cyanobacteria (blue-green algae) have a CO_2_-concentrating mechanism (CCM) which provides a growth advantage under conditions of limiting inorganic carbon (Ci; principally CO_2_ and HCO_3_^−^ in aquatic environments). The CCM has two functional features of note: the presence of a set of transcriptionally and post-translationally regulated Ci uptake systems which accumulate Ci as a cytoplasmic bicarbonate pool [1]; and the carboxysome, an icosahedral, protein-bound micro-compartment in which the action of carboxysomal carbonic anhydrase (CA) enzymes provides a CO_2_-rich micro-environment that supports the carboxylation reaction of d-ribulose 1,5-bisphosphate carboxylase/oxygenase (RuBisCO; EC: 4.1.1.39), thereby reducing the wasteful oxygenation reaction [2], [3]. Thus the concerted action of the cyanobacterial CCM improves photosynthetic rate under Ci limitation and reduces the energetic impact of the photorespiratory pathway.

Two evolutionarily distinct forms of carboxysome exist for which two monophyletic groups of cyanobacteria are named [4]–[6]. The *cso*-type α-carboxysome is found in α-cyanobacteria and probably first arose in chemo-autotrophic bacteria [4], [7], whereas the *ccm*-type β-carboxysome is found only in β-cyanobacteria [4], [6]. α- and β-carboxysomes appear to have evolved in parallel [5]. Whilst some of their constituent small proteins bear marked similarity to each other, other larger structural proteins have no observable similarity [2]–[4]. Specifically, the RuBisCO enzyme and the proteins predicted to form a semi-permeable shell structure are each distinct lineages of the same evolutionarily-related proteins [4], [6], [8], [9].

Whilst details of the interior structure of α-carboxysomes are scarce, these bodies are known to contain form-1A RuBisCO as well as a number of shell-associated proteins [10] including that presumed to organise the interior, CsoS2 [11], and the α-carboxysomal CA enzyme, CsoSCA [12], [13]. In contrast, the interior structure of β-carboxysomes is better understood [2], [3], [14]. In *Synechococcus elongatus* PCC 7942 and *Synechocystis* PCC 6803, an inner-shell bicarbonate dehydration complex and an interior para-crystalline matrix of form-1B RuBisCO molecules are linked and organised by at least two isoforms of the CcmM protein [15]–[17]. Current models of β-carboxysome organisation place a semi-permeable shell structure on the cytoplasmic face of a second, sub-shell, layer which contains the carboxysomal carbonic anhydrase enzymes [3], [14]–[19], thus two functionally distinct shell layers are present in β-carboxysomes: the semi-permeable shell layer and the bicarbonate dehydration complex/RuBisCO organising layer.

The outer shell layer of bacterial micro-compartments is thought to consist of oligomeric proteins containing the Bacterial Micro-Compartment (BMC) domain (pfam: PF00936) with the first known sequence for this family originating from *S. elongatus* PCC 7942 [20]. In crystal structures, BMC protomers form flattened hexagonal oligomers (hexamers or trimers of proteins containing one or two BMC domains respectively) which themselves tessellate into sheet or strip-like higher-level oligomers [18], [19], [21]–[27]. Current structural models suggest that these sheets and/or strips of BMC oligomers form the facets of the icosahedral carboxysome, the vertices being closed by pentameric proteins that contain a distinct type of protein domain (pfam: PF03319) [9], [19], [28], [29]. Charged pores at the six-fold axis of symmetry of BMC proteins are thought to underlie the selective permeability of the outer carboxysome shell [9], [30]. In addition to their potential as pores for charged solute transit, some BMC proteins have a complex pore conformation in crystal structures, evoking the potential for gated transit of larger metabolites [26]. On the other hand, some BMC proteins from related types of micro-compartment involved in ethanolamine and propanediol detoxification have absent or obfuscated pores [21]–[23], [25].

In *S. elongatus* PCC 7942, multiple low-mass bands corresponding to BMC proteins are sometimes observed in SDS-PAGE and western immunoblots, though there is a single band under normal conditions [15]. In α-cyanobacteria however, multiple shell proteins are evident in SDS-PAGE and western immunoblots [10], [12], [31], thus the complexity of the putative outer-shell complex appears to differ between carboxysome type and experimental conditions.

Significantly, cyanobacterial genomes can contain up to nine genes whose products contain a recognisable BMC domain (Table S1), however the specific roles that each of these proteins play, if any, is unclear [9]. Recent evidence suggests that simple micro-compartments can arise from the expression of a single BMC gene [32], so the shell structures of carboxysomes are not necessarily complex. Why then, do all cyanobacteria have between three and nine BMC homologues? It is well known that genes in the ‘core’ *ccm* operon *ccmK2-O* are essential for biogenesis of β-carboxysomes in *S. elongatus* PCC 7942 [20], [33]–[42]. Of these, *ccmK2* and *ccmO* have obvious BMC domains, indeed the β-cyanobacterial model *S. elongatus* PCC 7942 contains four genes with products containing BMC domains, *ccmK2* (named for sll1028 from *Synechocystis* PCC 6803, previously referred to as *ccmK* or *ccmK1*), *ccmK3*, *ccmK4* and *ccmO*, with the latter containing two BMC domains in tandem. Of this group, only CcmK2 has been observed in β-carboxysome-enriched fractions [15], [16], [43], thus other potential BMC proteins have no predicted functional role. Whilst fluorescently tagged CcmK4 proteins were shown to localise to the carboxysome [44], neither *ccmK3* nor *K4* have been implicated in carboxysome function or biogenesis in the model organism *S. elongatus* PCC 7942. Intriguingly, transposon mutagenesis of *ccmK4,* containing a single BMC domain, resulted in a strain requiring high-CO_2_ for growth in the related freshwater β-cyanobacterium *Synechocystis* PCC 6803 [45].

The ultrastructural phenotypes of β-carboxysome shell mutants are poorly understood. Mutant carboxysomes lacking the CcmL protein have a characteristic rod-like appearance which is consistent with its location at the icosahedral vertex in structural models [20], [34], [36]. Studies of the *ccmO* mutant N1 yield inconsistent results with some groups reporting the lack of β-carboxysomes [41], whereas others report occasionally aberrant carboxysomes [42]. An interesting carboxysome ultrastructure is apparent in *ccmN* mutants where a single polar carboxysome-like body is observed [33], [40], the proposal being that CcmN is required to attach the shell to the inner structures. Carboxysome-like polar bodies have been previously reported in a number of partially characterised mutants [38]–[40] and these were shown to contain all of the cells RuBisCO by Friedberg *et* *al.* [39].

No systematic study has investigated the BMC genes of any β-cyanobacterial species. With respect to other β-cyanobacterial species, *S. elongatus* PCC 7942 appears to have a relatively simple set of carboxysome genes. In light of recent insights into the potential structure of the outer carboxysome shell, each of the known BMC proteins from *S. elongatus* PCC 7942 was investigated by the construction of individual and double gene inactivation mutants. The carboxysome ultrastructure and function, physiology, growth rates, and protein components of carboxysomes were investigated in the mutant strains. Our main finding is that CcmK2 and CcmO are the predominant proteins in the outer shell of β-carboxysomes from *S. elongatus* PCC 7942, and may be structurally interactive, whereas CcmK3 and CcmK4 are probably rare but essential for high Ci-fixation rates in Ci-limited conditions.

## Results

Following initial growth testing, the carboxysomal ultrastructure of genetic mutants in BMC genes was investigated by transmission electron microscopy, and the protein components of wild-type and mutant β-carboxysomes identified there were further studied by carboxysome purification and western immunoblot analysis. To examine the physiological consequence of β-carboxysome shell perturbation the same strains were also assessed for their photosynthetic affinity for Ci and growth rates under Ci-replete and limited conditions. To maintain consistency with prior literature, the partially characterised HIND insertional mutant of *ccmK2* [20] was subjected to the same analyses but does not form a major part of the analyses presented here. In light of the similarity of the Δ*ccmK3* and Δ*ccmK4* mutants to wild type and their lack of a high-CO_2_ requiring phenotype, these genes were subsequently deleted in tandem. The resulting double-deletion mutant, Δ*ccmK3-4*, was subjected to the same analyses and, as described below, was deficient in some aspects of its CCM.

### Ultrastructural phenotypes of carboxysome shell mutants

**Figure 1:**
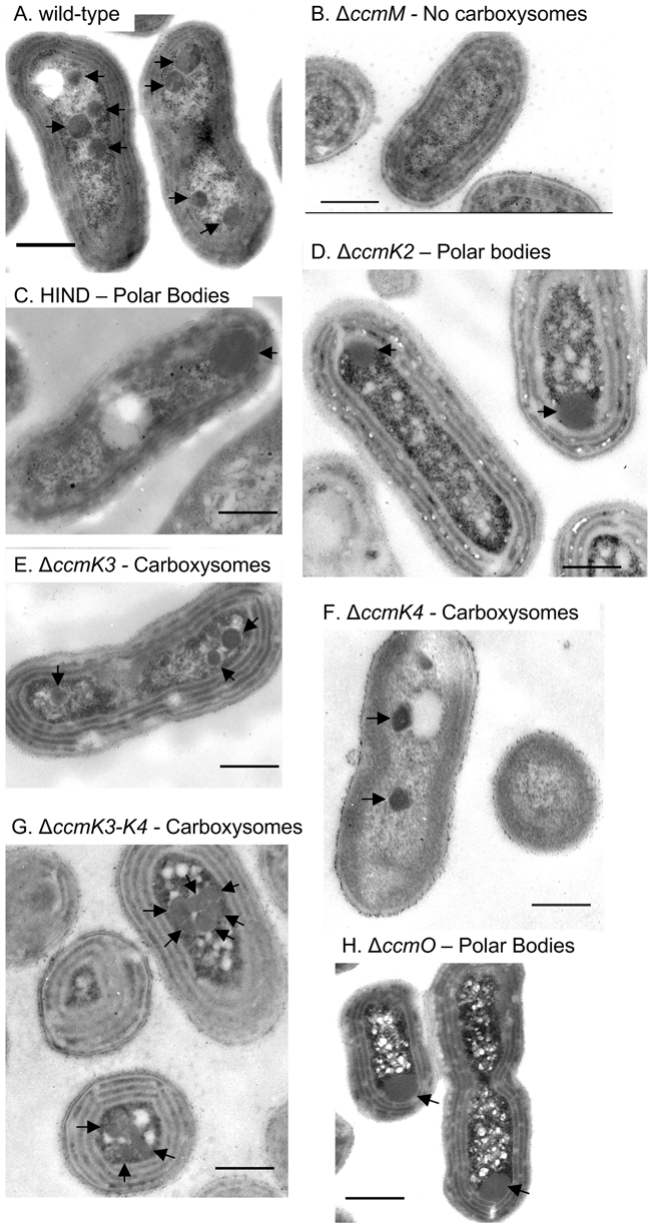
Ultrastructural carboxysome phenotypes in BMC shell mutants of *S. elongatus* PCC 7942. Representative transmission electron micrographs of each mutant generated here are shown, as well as the carboxysomeless Δ*ccmM* mutant for reference [16], [37]. **A**, Wild-type PCC 7942. **B**, PCC 7942 Δ*ccmM*. **C**, HIND mutant *ccmK2::CmR* [20]. **D**, Δ*ccmK2*. **E**, Δ*ccmK3*. **F**, Δ*ccmK4*. **G**, Δ*ccmK3-4*. **H**, Δ*ccmO*. Scale bars are 500 nm and HIND and Δ*ccmK4* were embedded in Epon-araldite rather than LR-white resin.

Transmission electron microscopy revealed two classes of ultrastructural phenotype in the mutants generated (Figure 1, Table 1), namely normal wild-type appearance or aberrant polar bodies. The wild-type strain had carboxysomes whose cross-sectional diameter (175±37 nm, Figure 1-A) compared well to previously published studies (172±26 nm [16]).

**Table 1:** Ultrastructural, physiological and growth parameters for BMC mutants.

	Carboxysome type and diameter		*K*_1/2_ (Ci) (mm)	Doubling time (h)		Physiology^B^
Genotype	Type^A^	Diameter (nm)		4% CO_2_	Air	
**PCC 7942**	CBX	175±37 (35)	0.27±0.05	8.6±0.4	10.7±0.6	WT
**HIND** C	PB	318±56 (4)	17.8±1.5	8.4±0.1	n.g.	HCR
**Δ** ***ccmK2***	PB	361±78 (56)	16.5±0.6	10.8±0.5	n.g.	HCR
**Δ** ***ccmK3***	CBX	167±26 (50)	0.34±0.04	9.0±0.3	10.0±0.2	WT
**Δ** ***ccmK4***	CBX	169±36 (33)	0.35±0.05	7.7±0.7	9.6±0.4	WT
**Δ** ***ccmK3-4***	CBX	183±24 (50)	10.0±2.3	10.0±1.5	28.9±0.9	HCR^D^
**Δ** ***ccmO***	PB	302±54 (56)	17.3±0.6	9.3±0.7	n.g.	HCR
**Δ** ***ccmM*** C	None	n.a.	19.2±2.38	8.1±0.4	n.g.	HCR

Carboxysome diameters are the mean and standard deviation of a sample size shown in brackets and photosynthetic half-saturation constant *K*_1/2_ (Ci) and growth rates are expressed as the mean and standard deviation of at least three replicate cultures.

A , Wild-type like carboxysomes (**CBX**) and carboxysome-like polar bodies (**PB**).

B , wild-type like (**WT**) or high CO_2_ requiring (**HCR**).

C , The partially characterised HIND mutant (*ccmK2::Cm^R^*) [20] and the carboxysomeless Δ*ccmM* mutant [37] are included for reference.

D , Δ*ccmK3-4* has an intermediate physiology which is essentially HCR.

**n.g.**, no growth.

**n.a.,** Δ*ccmM* has no carboxysome ultrastructure.

Like the wild type strain, the Δ*ccmK3* and Δ*ccmK4* mutants possessed carboxysomes whose appearance and dimensions compared favourably to wild-type (167±26 and 169±36 nm respectively, Figure 1-E, F). The Δ*ccmK3-4* mutant had carboxysomes with a mean diameter of 183±24 nm. However, the spatial arrangement of carboxysomes within the cell was altered in this strain with carboxysomes aggregated (Figure 1-G) rather than well-separated as observed in wild-type cells (Figure 1-A).

In contrast to the wild type-like mutants, the HIND, Δ*ccmK2* and Δ*ccmO* mutants possessed large, polar, electron-dense structures which lacked the characteristic faceted geometry of carboxysomes (Figure 1-C-D, H, Table 1). The maximum cross-sectional diameter of these bodies was much greater than wild-type, though still smaller than wild-type β-carboxysomes from some β-cyanobacterial species [46].

Occasional aberrant carboxysomes were observed in a complemented mutant strain generated in this study, Δ*ccmO* + pH6-Ub-*ccmO* (Figure S1-C), however, the majority of carboxysomes in this strain, whilst being slightly larger than wild-type (208±49 nm), appeared normal and the strain was not physiologically impaired (Figure S2). Nonetheless, occasional aberrant carboxysomes observed in this strain included rod-like carboxysomes reminiscent of those described for the PVU mutant (*ccmL::Cm^R^*) [20], [36].

### Physiological phenotypes of BMC mutants

**Figure 2:**
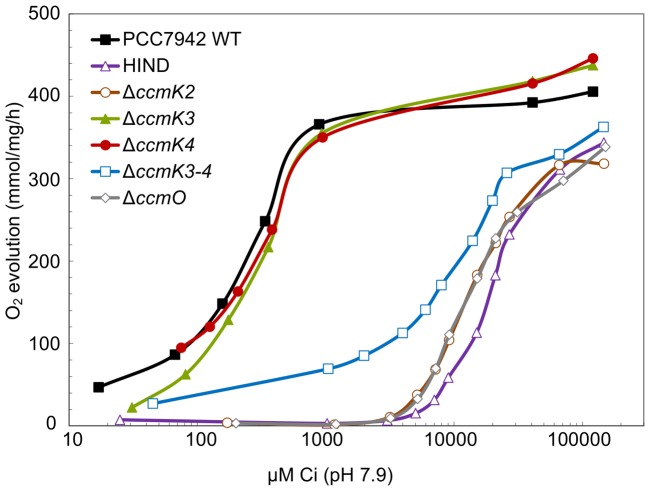
Photosynthetic oxygen evolution in response to external Ci by BMC shell mutants. Shown are a representative set of mass-spectrometric measurements of Ci-dependent O_2_ evolution by wild type and mutant strains of *S. elongatus* PCC 7942 over a range of Ci concentrations. Ci is the sum of CO_2_ and HCO_3_^−^ in solution (pH 7.9).

Although two types of carboxysome ultrastructure were observed, three classes of physiological phenotype were apparent within the mutants generated in this study (Table 1, Figure 2). Mutants were assessed for differences in photosynthetic affinity for Ci (*K_1/2_* Ci) derived from oxygen evolution curves plotted against Ci concentrations [47]. The Δ*ccmK3* (*K_1/2_* (Ci) 0.35±0.05 mm) and Δ*ccmK4* (0.34±0.04 mm) mutants had Ci-uptake physiology that closely matched the wild-type strain (0.27±0.05 mm). In contrast, the polar body mutants HIND (17.8±1.5 mm), Δ*ccmK2* (16.5±0.6 mm) and Δ*ccmO* (17.3±0.6 mm) had very highly attenuated CCM activities (Figure 2), which agreed with the physiological findings of previous studies of *ccmK2* [20] and *ccmO* mutants [41], [42]. Indeed, the photosynthetic physiology of these mutants was similar to Δ*ccmM* which completely lacks carboxysomes [16]. A third, unusual phenotype was observed for the Δ*ccmK3-4* mutant which displayed a photosynthetic physiology that was intermediate to the wild-type and carboxysome mutants (10.0±2.3 mm; Figure 2). Hence, although essentially a high-CO_2_ requiring mutant, Δ*ccmK3-4* could photosynthesise and grow in air.

These physiological findings were supported by the comparative growth rate analysis of these mutant strains under conditions of high and low CO_2_ (Table 1). All strains grew at a similar rate under 4% CO_2_ where the doubling times ranged from 7.7±0.7 to 10.8±0.5 h. The wild-type, Δ*ccmK3* and Δ*ccmK4* strains also grew at a similar rate in air (9.6±0.4 – 10.7±0.6 h). However, the carboxysome mutants HIND, Δ*ccmK2* and Δ*ccmO* were not capable of growth without Ci supplementation. Like its Ci-uptake phenotype, the Δ*ccmK3-4* mutant grew in air, albeit very slowly (doubling time 29.9±0.9 h).

### Protein composition of isolated β-carboxysome preparations

The protein components of carboxysomes and polar bodies were assessed by western immunoblots against protein extracts enriched in carboxysomes using the Mg^2+^ precipitation and Triton X-100-Percoll (TP pellet) methods [15], [36], [43], [48], [49]. Since approximately 73% of CcmK2 remains in the insoluble pellet fraction after cell lysis [14], the Triton X-100-Percoll purification was appropriate for purification of carboxysome-like polar bodies because the effectiveness of this method is independent of the presence of an intact native outer shell structure. As described below, the outcomes of both Triton X100-Percoll purification closely matched those from Mg^2+^ precipitation which is a structure independent method for visualising carboxysomal proteins, thus validating these assumptions.

**Figure 3:**
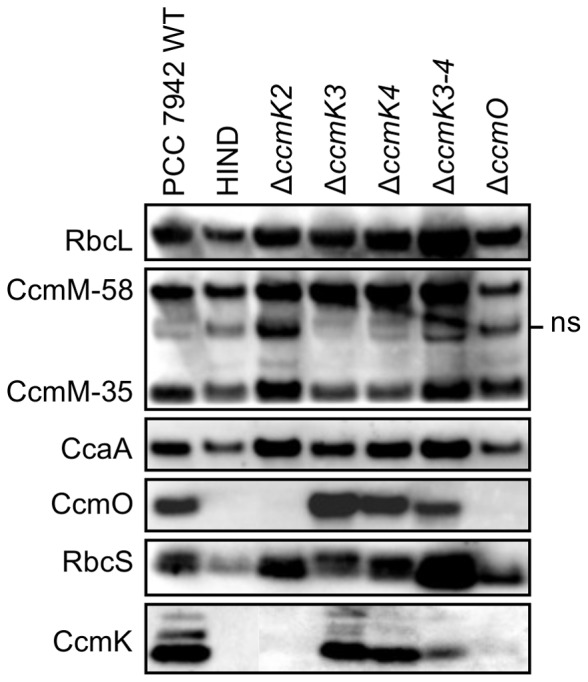
Carboxysomal proteins in wild-type *S. elongatus* PCC 7942 and BMC mutants. Western immunoblots show the presence or absence of carboxysomal proteins in carboxysomes purified to the Triton X-100-Percoll stage of the Epps-EDTA method for β-carboxysome purification [43], [48]. Polyclonal antibodies against CcaA, RbcLS, CcmM-35, CcmK2, and CcmO were used [15]. The CcmM-35 antiserum detects CcmM-58 and CcmM-35, as well as a non-specific band (**ns**) corresponding to RbcL that is routinely visible in CcmM western immunoblots [15]. The CcmK2 antibody detects all three CcmK homologues, but not CcmO, whereas the CcmO antibody detects CcmO specifically.

The pattern of carboxysome protein presence and absence from TP pellets (Figure 3), and Mg^2+^ fractions (Figure S3), was similar for all strains. In terms of the inner shell bicarbonate dehydration layer and carboxysome lumen, all detectable components of the RuBisCO organising complex (CcaA, CcmM-58, CcmM-35, and RuBisCO) were present in carboxysomes and polar bodies (Figure 3, Figure S3), however the presence of CcmN could not be reliably confirmed using our low-titre antibody. The 35 kDa form of CcmM, which predominantly links RuBisCO holoenzymes into an interior matrix, was present in the carboxysome enriched fractions of all of the mutants, even those possessing polar bodies.

CcmK2 *and* CcmO were not detected in TP pellets of *ccmK2* and *ccmO* mutants respectively, and a relationship was shown between CcmK2 and CcmO such that the absence of one of this pair resulted in the absence of the other (Figure 3). This pattern was in-part repeated in Mg^2+^-precipitated fractions, where the presence of CcmO was dependent on the presence of CcmK2 without the reverse situation being true (Figure S3). Intriguingly, for these mutants the CcmO or CcmK2 proteins were not found in the Mg^2+^ supernatant. Thus CcmO, like CcmK2 [14] may largely be lost from the shell and aggregated after cell breakage, especially in the absence of other shell proteins. This is the first evidence of a valid structural interaction between CcmK2 and CcmO in the β-carboxysome and is not that surprising given that these proteins share the BMC protein domain.

### IMAC purification of interacting BMC shell proteins

**Figure 4:**
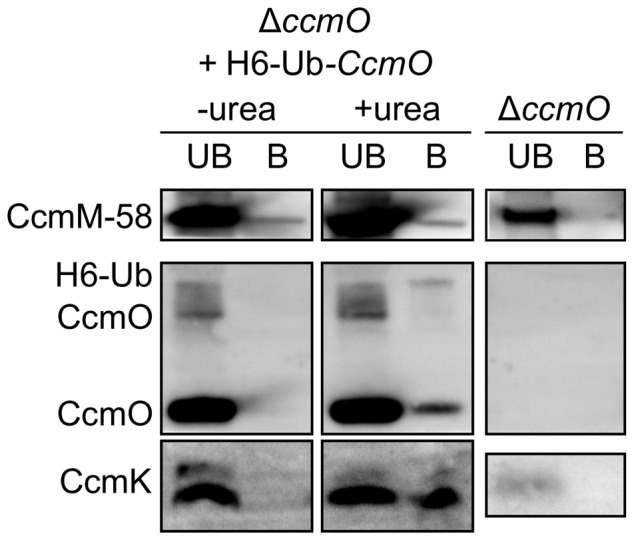
Protein:protein interactions between CcmO and CcmK2. Western immunoblots showing carboxysome components IMAC-purified with H6-Ub-CcmO from TP pellets in the presence and absence of 1.0 m urea (**+/−urea**). **UB**, IMAC unbound fraction. **B**, IMAC bound fraction. The presence of a small amount of CcmK in the Δ*ccmO* mutant is attributed to the very high loading of these SDS-PAGE gels in comparison to those presented in Figure 3. The specificities of the different antibodies are explained in the caption to Fig. 3.

To investigate the observed relationship between CcmK2 and CcmO in the shell of β-carboxysomes (Figure 3), immobilised metal affinity chromatography (IMAC) was used to IMAC-purify hexahistidine tagged CcmO proteins along with any protein-interaction partners from Δ*ccmO +* pSE2-H6-Ub-CcmO (Figure 4). To enhance resolution of complexed proteins, the analysis was pre-enriched for carboxysome proteins by using TP pellets prepared from Δ*ccmO* + pSE2-H6-Ub-CcmO as a substrate for IMAC. Tagged and complexed proteins were identified by western immunoblots. As observed by Long *et* *al.* [16], partial denaturation with 1.0 m urea was required for IMAC co-purification of β-carboxysome protein complexes. This confirms previous work showing that BMC proteins like CcmK2 and CcmO form extremely stable oligomers which often cannot be dissociated, even with high urea concentrations [14]–[16]. Thus a small amount of urea is necessary to sufficiently solubilise or weaken the BMC protein oligomers, allowing effective purification of these proteins and their binding partners from the outer shell of β-carboxysomes.

CcmK2 was confirmed as an *in vivo* protein binding partner of CcmO (Figure 4) as suggested by the western immunoblot analysis above (Figure 3). It was not clear whether this was due to the presence of hetero-oligomers formed from CcmK2 and CcmO or interactions between adjacent protein oligomers in the β-carboxysome shell. The untagged form of CcmO was the predominant form co-purified from TP pellets of Δ*ccmO* + pH6-Ub-*ccmO* (Figure 4). Complicated expression of both tagged and untagged forms of H6-Ub chimeric constructs are well known [50], [51] explaining the presence of both forms of recombinant CcmO in the TP pellet. Significantly, that untagged CcmO was co-purified by H6-Ub-CcmO confirms speculation that CcmO forms oligomeric proteins like other BMC proteins.

The small amount of CcmM-58 detected in the IMAC bound fraction (Figure 4) was probably not due to an interaction between CcmO and CcmM-58. A similar amount of CcmM58 is present under both urea treatments (Figure 4). This is hardly surprising given that CcmM-58 is an abundant cellular protein [52], and is certainly one of the most abundant proteins in TP pellets [16], [43] suggesting non-specific carryover of CcmM-58 into the IMAC eluate. Nonetheless, the presence of CcmM-58 in the eluate would not be unexpected given its proven interaction with CcmK2 [17].

## Discussion

### CcmK2 and CcmO are the predominant proteins of the outer β-carboxysome shell in *S. elongatus* PCC 7942

The outer shell of β-carboxysomes is predicted to be formed from a number of subtly different CcmK homologues [9]. In *Synechocystis* PCC 6803, CcmK1 and CcmK2 have been postulated as major shell proteins whereas CcmK3 and CcmK4 are probably minor [29]. Of these, *S. elongatus* PCC 7942 has the *ccmK2, K3, K4* and *ccmO* genes. We argue that CcmK2 and CcmO are the major proteins, and CcmK3 and CcmK4 are minor proteins forming the outer shell of β-carboxysomes from this strain.

In this report, we showed that some BMC mutants formed aberrant carboxysome ultrastructure. The polar body mutants HIND, Δ*ccmK2* and Δ*ccmO* were phenotypically high-CO_2_ requiring and thus incapable of growth in air (Table 1). Previously, some partially characterised *ccmN* and RuBisCO small subunit mutants have exhibited the polar body carboxysome phenotype [33], [39], [40], and these polar bodies were shown to contain most, if not all of the RuBisCO of the cell [39]. Interestingly, the ultrastructure of Δ*ccmO* carboxysomes presented here (Figure 1-H) contradicts previous analyses of *ccmO* mutants which revealed either no carboxysomes [41], or a large proportion of aberrant carboxysomes [42]. We suggest that our specific genetic deletion provides a better experimental model than the insertional *ccmO* mutant investigated previously, hence the phenotypic difference.

The structural basis for polar bodies was clear. It was apparent that a structurally relevant interaction between CcmK2 and CcmO represents a key interaction for the integrity of the β-carboxysome outer shell (Figure 3, Figure 4). Previous work showed no interaction between CcmO and CcmK2 [17] but we showed that genetic deletion of either of the genes abolished not just that protein, but also the other from carboxysome-enriched TP pellets (Figure 3), and these were subsequently shown to interact *in vivo* (Figure 4). In contrast to the proteins identified from TP pellets (Figure 3), very small amounts of CcmK2 were present in the Mg^2+^ pellet from the Δ*ccmO* mutant (Figure S3). This may indicate the presence of a partial, or incomplete shell structure on polar bodies in these mutants, or the spontaneous and extraneous association of outer shell sub-complexes to the polar body due to the well-established interaction between CcmK2 and CcmM [15]–[17]. Indeed, polar-body mutants had high CO_2_-requiring (HCR) physiological phenotypes which were similar to that of the carboxysomeless Δ*ccmM* mutant (Figure 2), indicating the lack of an effective shell structure.

### Polar bodies are ordered structures containing active RuBisCO

In terms of the inner structure of polar bodies, western immunoblot against known β-carboxysome components showed that polar bodies contained all of the protein components expected of β-carboxysomes except the major outer shell proteins (Figure 3). Indeed, the maximum cross-sectional diameters of polar bodies (as well as carboxysomes) increased consistently as expected if these bodies were to contain regularly increasing RuBisCO layers bound together by CcmM-35 [14]. It is well known that cyanobacteria require high RuBisCO activity to survive, and because polar bodies were previously shown to contain all of the RuBisCO of the cell [39]. Thus in contrast to the supposition of Kinney *et* *al.* [33], we argue that polar bodies are physiologically relevant, ordered structures containing active RuBisCO. During β-carboxysome biogenesis there must therefore be a mechanism by which nascent carboxysome-like complexes are constrained into smaller carboxysomes rather than polar bodies. Kinney *et* *al.* [33] proposed that the CcmN protein is important for this process, and that this protein could bridge the inner and outer shell layers. Based on the shared ultrastructure of *ccmK2, ccmO* and *ccmN* mutants we agree that this is a reasonable proposal.

So far, no α-carboxysome mutant has been reported that results in polar bodies, perhaps stemming from their relative lack of internal structure and the less complete set of genetic knock-outs so far completed. However, within the propanediol utilisation micro-compartment, which has eight putative shell proteins, the Δ*pduB′* mutant results in polar bodies. These bodies are much smaller than those reported here, and they probably represent aggregated active protein components from the PDU compartment [53]. The shell proteins identified in PDU micro-compartments are varied, and two protein products of *pduB′* make up approximately 25% of the PDU micro-compartment [54]. Thus polar body formation after disruption of the major shell proteins could be a common feature of bacterial micro-compartments, and not necessarily due to general protein aggregation.

### CcmK3 and CcmK4 are minor outer-shell components

In contrast to the HCR mutants containing polar bodies, the remaining mutants, Δ*ccmK3,* Δ*ccmK4* and Δ*ccmK3-4,* had wild-type like carboxysomes (Figure 1) but those from Δ*ccmK3-4* were physiologically impaired (Figure 2). The phenomenon of physiologically impaired carboxysomes with normal ultrastructure is not novel, as α-carboxysomes lacking the vertex proteins CsoS4A and CsoS4B were shown to have increased CO_2_ leakage from ostensibly normal carboxysomes [28]. In terms of their protein composition, comparison of the CcmK western immunoblot diagnosed the absence of detectable quantities of CcmK3 and CcmK4 from TP pellets (Figure 3). This suggests that multiple CcmK bands, observed here and previously [15], may be breakdown or alternative products of CcmK2 as they are only abolished by the *ccmK2* and *ccmO* deletions (Figure 3). Therefore the protein evidence is consistent with CcmK3 and CcmK4 being low-abundance carboxysome proteins. The physiology and ultrastructure of β-carboxysomes from Δ*ccmK3-4* support this claim. This is not unexpected given their BMC domain and their Ci-responsive co-expression with other carboxysome components [55], [56] Indeed, Savage *et* *al.* [44] showed that fluorescently labelled CcmK4 localised to the β-carboxysome however no previous studies have studied the sub-cellular localisation of CcmK3. Further, because the tandem deletion of *ccmK3-4* is required to produce an observable phenotype, it is likely that the structural and functional roles of CcmK3 and CcmK4 are redundant in β-carboxysomes. In addition, because the CcmK4 protein is rare, and no ultrastructural phenotype was detected in the Δ*ccmK4* mutant, we propose that the linear structures observed in crystallographic studies of CcmK4 [18] may be artefacts of crystallisation and are unlikely to represent native carboxysomal protein complexes.

The potential functions of CcmK3 and CcmK4 may include niche structural roles within the outer shell such that they close the outer-shell rather than form the bulk facet. CcmK3 and CcmK4 could also have a role in channelling RuBisCO substrates or co-factors through their central pores. Of these alternatives, the Ci-uptake physiology of Δ*ccmK3-4* is more consistent with the unclosed shell hypothesis. The likely physiological phenotype of carboxysomes with reduced solute transit would imitate the wild-type response to dissolved Ci, the maximum photosynthetic rate being limited by the availability of that particular substrate.

### CcmK3 and CcmK4 are required for correct subcellular localisation of β-carboxysomes

Recently, the spatial arrangement of β-carboxysomes was shown to depend on the bacterial cytoskeleton [44]. The precise interactions underlying β-carboxysome localisation and partitioning are unclear, however the cytoskeleton proteins ParA and MreB are essential in *S. elongatus* PCC 7942 [44] and we present evidence in this report that CcmK3 and CcmK4 are individually required for correct subcellular localisation of β-carboxysomes (Figure 1-G). The phenotype of seemingly normal, aggregated, micro-compartments was also observed in the *pduK* mutant of *Salmonella enterica*, whose PDU micro-compartments are involved in degradation of 1,2-propanediol [53]. This suggests a common mechanism for arrangement and partitioning of different types of bacterial micro-compartment across vast phylogenetic distances.

Aberrant carboxysome partitioning is insufficient to explain the physiological phenotype of the Δ*ccmK3-4* strain. Savage *et* *al.* [44] showed that the *parA* mutant was unable to correctly apportion β-carboxysomes within the cell, and to daughter cells at mitosis. However this strain had only a very slight growth rate disadvantage whereas the Δ*ccmK3-4* mutant had a severely reduced growth-rate in air. We perceive similar implications for carboxysome partitioning during mitosis in the polar body mutants HIND, Δ*ccmK2* and Δ*ccmO*. Supporting this, numerous cells without obvious PBs were observed in TEM sections, indicating that the PBs, and thus the cellular RuBisCO, were not partitioned to daughter cells in the same even manner as in β-carboxysomes in wild-type cells. Thus we reason that for the mutants with polar bodies, half of the daughter cells at mitosis will inherit all of the RuBisCO of the parental cell. Hence some part of the HCR phenotype of HIND, Δ*ccmK2* and Δ*ccmO* is probably caused by the lack of recruitment of CcmK3 and CcmK4 to the carboxysome, and subsequent deficiencies in localisation and inheritance of carboxysomes during mitosis. This is perhaps an extreme example of the type of β-carboxysome partitioning deficit described by Savage *et* *al.* [44].

### Variable numbers of *ccmK* genes

We have shown that in *S. elongatus* PCC 7942 there are two major β-carboxysome shell components, CcmK2 and CcmO, and two minor, CcmK3 and CcmK4. Thus the minimum BMC-gene requirement for a structurally relevant carboxysome is *ccmK2* and *ccmO*, whereas accessory BMC genes such as *ccmK3-4* refine the functionality of the shell. Intriguingly, other β-cyanobacteria have as many as nine identifiable BMC genes and most of the variability in β-cyanobacterial BMC genes is due to accessory minor shell proteins (Table S1). The potential for CcmK3 and CcmK4 proteins to act on carboxysome localisation and partitioning suggests that the variable BMC gene complement reflects the varied morphology of cyanobacterial cells. Perhaps the wide variation in cyanobacterial morphology and subcellular structure is matched by a varied cytoskeletal structure and dynamic, thus the carboxysome must be able to flexibly alter its shell structure in order to maintain cognate interaction with a similarly varied or flexible cytoskeleton. However there is no obvious correlation between number of accessory *ccmK* genes and lifestyle or environment niche of β-cyanobacterial strains.

In *Synechocystis* PCC 6803, *ccmK4* was previously shown to be essential for photoautotrophic growth [45]. Thus there are questions over the roles that accessory CcmK proteins play in different β-carboxysome systems. One wonders what aspect of *Synechocystis* carboxysomes makes CcmK4 an essential protein. It is possible that the multiple transposon mutants of *ccmK4* identified by Zhang *et* *al*. [45] were also polar mutants of *ccmK3*. This seems implausible, leading to the conclusion that different *ccmK* homologues have different functions, or are under different selective pressures in different β-cyanobacteria.

### Does the CcmO protein recruit the vertex pentamer CcmL?

CcmL is predicted to occupy the vertices of β-carboxysomes [19] and is required for effective shell function. Its absence leads to rod-like carboxysomes in β-cyanobacteria [20], [34], [36]. Rod-like and potentially tetrahedral carboxysome-like inclusions were sometimes observed in the Δ*ccmO* + pH6-Ub-*ccmO* complemented strain (Figure S1), hinting at the possibility of a structural interaction between CcmO and CcmL. As evidenced by protein analysis, the predominant CcmO form in carboxysome-enriched TP pellets was the untagged form (Figure 4) which probably arises from some proteolytic activity *in vivo*. Hence two possibilities may underlie the occasional occurrence of structural aberration: Structural perturbation caused by the 11 kDa hexahistidine-ubiquitin tag itself, or an improper stoichiometry between shell proteins due to the non-native promotor from which the tagged *ccmO* gene was expressed.

The rod-like carboxysome phenotype is usually attributed to a syndrome of CcmL insufficiency where the vertices of the carboxysome cannot close. Thus, if CcmL is recruited to the β-carboxysome by CcmO, and the quantity of CcmO is sub-stoichiometric due to expression from the non-native promotor, the result may be a low rate of mutant carboxysomes that resemble the authentic *ccmL* mutant. Alternatively, the CcmO protein may tolerate some forms of modification; indeed it has variable N- and C-terminal domains which make it the least conserved BMC protein in β-cyanobacteria. Based on these data, we would argue that CcmO interacts with CcmL at the vertices of the carboxysomal icosahedron. Indeed, it is possible that CcmO forms a possible bridging protein, or “zipper”, that helps fix the interface between neighbouring outer-layer triangular facets.

### CcmP: potential RuBP pore for the β-carboxysome outer shell?

The α-carboxysomal BMC gene *csoS1D* was recently shown to have a β-cyanobacterial homologue, *ccmP* [56]. This gene was not obviously related to the β-carboxysome at the outset of this study, thus it is absent from the analysis presented here. Nonetheless it is an obvious focus for future work, the α-carboxysomal homologue CsoS1D was shown to be exceedingly rare in the isolated α-carboxysomes of *Prochlorococcus* sp. str. MED4, existing at less than one functional unit per α-carboxysome facet [31]. This protein was also shown to be important, but not essential for correct α-carboxysome ultrastructure in an ectopic α-carboxysome expression study [57]. Early assertions that the CsoS1D protein may form a gated pore for RuBP [26] seem not to be borne out by experimental studies where its role appeared to be primarily structural [57]. Thus it remains to be seen whether the structure of β-carboxysomes is dependent on the protein product of *ccmP*.

### A tentative model for the interaction of outer shell proteins in *S. elongatus* PCC 7942

Given the carboxysome ultrastructure and physiology of Δ*ccmK3-4*, it appears that as few as three proteins, CcmK2, CcmO and CcmL, form the minimum set required to construct an outer shell that is capable of supporting photo-autotrophic growth in air levels of CO_2_ –bearing in mind that an outer shell cannot form without the 35 and 58 kDa isoforms of the RuBisCO-organising CcmM protein [15], [16], [17]. Based on observed structures in some Δ*ccmO* + pH6-Ub-*ccmO* cells the vertex protein CcmL could interact with CcmO, perhaps suggesting a structural role for the CcmO protein at the vertices of the carboxysome. The CcmK2 protein is known to interact with the inner shell proteins CcmM-58 and CcmN [15], [17], and is likely to form the bulk of the facets. CcmK3, CcmK4, and potentially CcmP, probably have niche roles, and CcmK3 and CcmK4 are individually required to close the carboxysome shell, potentially at very specific locations.

**Figure 5:**
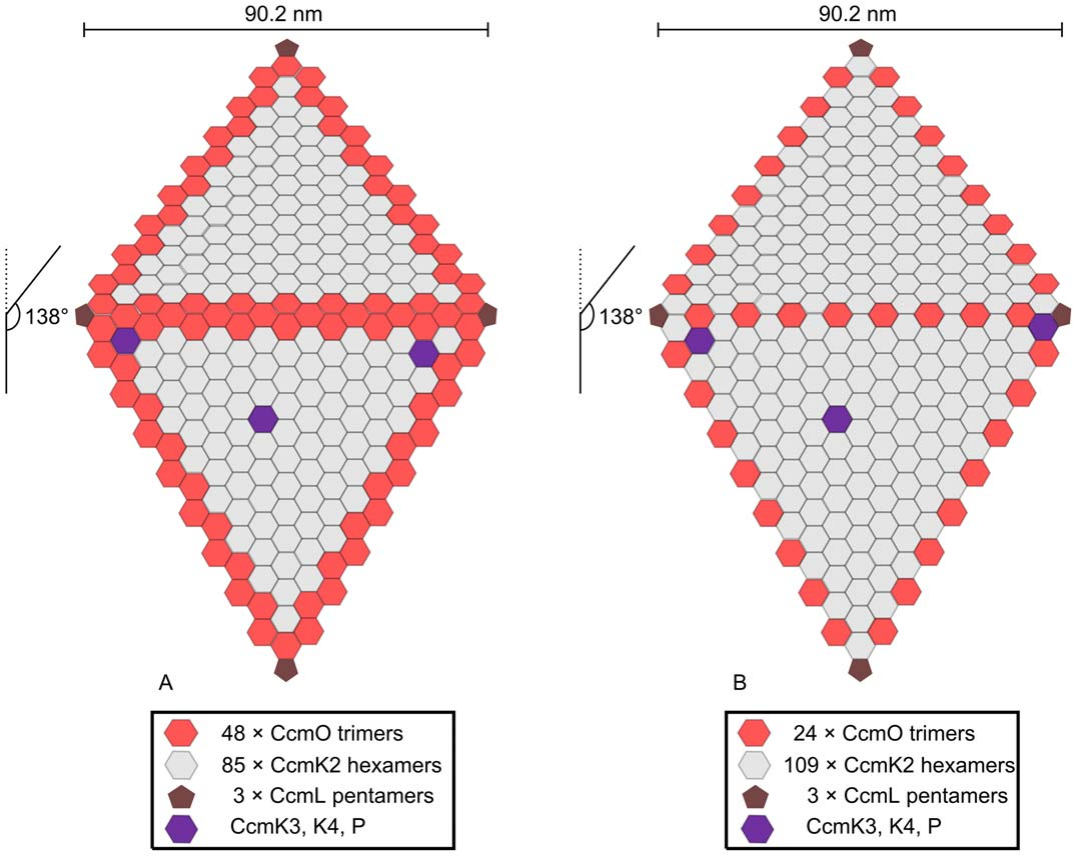
Two models for the outer shell facets of β-carboxysomes in *S. elongatus* PCC 7942. **A**, a β-carboxysome facet model where the entire edge structure is formed from CcmO. **B**, a β-carboxysome facet where CcmO only occupies the EutS-like role. Two facets are shown with the top facet extending into the plane of the drawing at 138° from the bottom facet. Structural roles are: edge (CcmO, red), facet (CcmK2, grey), vertex (CcmL, brown) and niche (CcmK3, K4 and CcmP, Purple). The number of individual protein oligomers forming a single facet is listed below. The carboxysome edge length (90.2 nm) was calculated as the number of CcmK2 hexamers (CcmK2 hexamer edge length  = 35 Å [9]) sufficient to account for the facet-edge length (92.0 nm) calculated from the maximum cross-sectional diameter reported here (175±37 nm). The maximum cross-sectional diameter was assumed to represent the radius of a sphere circumscribing the icosahedral carboxysome.

The data presented in this study suggest potential models describing the outer-shell structure of facets in idealised β-carboxysomes from *S. elongatus* PCC 7492 (Figure 5). The first facet model for the outer-shell of idealised β-carboxysomes (Figure 5-A) has CcmK2 as the predominant protein of the β-carboxysome facet, covering ∼70% of the facet surface (Table 2). CcmO is shown at the vertex-facet interface as well as the facet-facet interface and covers ∼30% of the surface. The second model (Figure 5-B) has CcmO trimers at defined locations along the edges of the β-carboxysome, as a ‘zipper’ protein that forms ∼10% of the β-carboxysome surface (Table 2).

**Table 2:** Protein components of the outer shell of idealised models of the β-carboxysome presented in Figure 5.

		CcmK2	CcmO	CcmL
**Model A**	**Multimers per carboxysome**	1700	720	12
	**Surface area (%)**	69.9	29.6	0.5
**Model B**	**Multimers per carboxysome**	2180	240	12
	**Surface area (%)**	89.6	9.9	0.5

The location of CcmO in both models (Figure 5-A,B) is supported by the observation that CcmO was not detected in the Mg^2+^ pellet of Δ*ccmK2* and HIND mutants, but that CcmK2 was detected at low levels in the Mg^2+^ pellet of Δ*ccmO* (Figure S3): CcmK2 can associate to the polar body through its dual interactions with CcmM and CcmN, but CcmO does not (Figure 4), probably because the opportunity for interaction with internal proteins is minimised at the facet-facet interface. Similarly, the stoichiometry of CcmK2 hexamers to CcmO trimers in model A (Table 2) is consistent with the observed amount of CcmK2 in β-carboxysomes, which by current estimates covers only 63–75% of the carboxysome surface in *S elongatus* PCC 7942 [14]. Thus we postulate that the amount of CcmO protein in model A is consistent with reported shortfall in β-carboxysome surface coverage.

Also, CcmO requires interaction with the CcmK2 proteins of the carboxysome facet for incorporation into the carboxysome. Thus we postulate that CcmO could form the entire edge structure and interfaces adjacent facets (Figure 5-A). This speculative model accounts for the minimum set of components encoding a functional outer shell structure CcmK2, CcmO and CcmL, and accounts for structural requirements at the facet-facet interface by assuming that CcmO is able to fill two structural roles: flat hexagonal trimers at the interface with CcmK2, and bent trimers at the interface between the facets. This assumption is supported by the behaviour of EutS which is able to adopt flat and bent conformations in the ethanolamine detoxification micro-compartment [23], [32].

Our alternative model where CcmO only fulfils the EutS-like bent-oligomer role (Figure 5-B, Table 2) is more economical with regard to CcmO content per carboxysome, and may be unsatisfactory with respect to observed amounts of CcmK2 proteins reported by Long *et* *al.* [14]. Nonetheless, the model B demands only a single oligomeric conformation for the CcmO protein – the ‘bent’ EutS-like conformation. To date, the exact nature of CcmO trimers has not been revealed by protein crystallography, thus the exact role played by CcmO in the outer β-carboxysome shell remains speculative.

## Materials and Methods

### Bacterial strains and culture conditions

*S. elongatus* PCC 7942 and mutant derivatives were maintained on modified BG-11 medium [58]–[60] solidified with 1.2% agar. The same medium was used for growth analyses and liquid cultures but for physiological analysis by membrane-inlet mass spectrometry NaNO_3_ was replaced with 20 mm NaCl and the medium was buffered with 50 mm bis-tris propane (pH 7.9).

Gene inactivation plasmids were generated in pUC18 which does not replicate in *S. elongatus* PCC 7492. Primers used for PCR are listed in Table S2. Gene inactivation plasmids were constructed in the pUC18 plasmid backbone as described previously [37] using a selectable chloramphenicol resistance marker (Cm^R^) [61]. DNA sequences of all constructs were confirmed by DNA sequencing and the final plasmids were transformed into *S. elongatus* PCC 7492 as described previously [34], [37]. Segregated transformants were confirmed by diagnostic PCR and restriction digestion, these strains are listed in Table S3. The DNA oligonucleotides used for PCR are listed in Table S4.

### Complementation of mutant strains

Phenotypically mutant strains were subsequently complemented by reintroduction of their respective genes from an *E. coli/S. elongatus* shuttle vector. *ccmK2* was reintroduced into HIND and Δ*ccmK2* under control of its native promotor in the vector pSE41 (derived from pSE4 [62], [63] in this work), *ccmO* was reintroduced into Δ*ccmO* under control of the *lac* operator in the pSE2 vector [64], and *ccmK3-4* were reintroduced into Δ*ccmK3-4* under control of their native promotor in the pSE41 vector. The pSE41 *E. coli/S. elongatus* expression vector was constructed by the in-frame insertion of the promotorless ampicillin resistance gene from pUC18 as well as additional synthetic restriction sites (NheI, BmtI, BglII, ScaI and XhoI) into the NcoI/XbaI restriction sites within the polylinker sequence of pSE4 [63]. The pSE41 plasmid is ampicillin and spectinomycin resistant in *E. coli*. The pSE41 plasmid map is shown in Figure S4, and the complemented strains are listed in Table S3. The DNA oligonucleotides used for PCR are listed in Table S4.

Previous studies have shown that effective IMAC purification of β-carboxysomal proteins was enhanced by insertion of a spacer domain between the protein and the hexahistidine tag (chloramphenicol acetyltransferase, CAT in [16]). In the current study we used the pHUE hexahistidine-ubiquitin tagging system to produce a single plasmid construct for both complementation of Δ*ccmO* and IMAC-purification of CcmO-interacting proteins [50], [51]. The ubiquitin tag has been shown to be a non-interacting tag that has been effective in the improved solubility of otherwise problematic proteins [50], [51].

The mutant strains that had detectable ultrastructural or physiological phenotypes were complemented to wild type carboxysome structure, arrangement and function, showing that the mutant phenotypes were entirely attributable to the specific gene deletions (Figure S1, Figure S2, Table S2).

### Physiological analyses

Physiological measurements by membrane-inlet mass spectrometry (MIMS) were performed as described previously [65], [66], and maximum growth rates were measured as described by Long *et al*. [16].

### Transmission electron microscopy

Cells were prepared for transmission electron microscopy (TEM) essentially as described previously [34] the exception being that the cells were embedded in LR-White resin or epon-araldite rather than Spurrs resin. Sections were stained with 2% uranyl acetate and Reynolds lead citrate. The sections were viewed in a Hitachi H7000 transmission electron microscope (Hitachi Ltd, Tokyo, Japan) at 75 kV. Measurement of carboxysome diameter was performed using ImageJ 1.45 [67]. Carboxysome measurements were made at the widest cross-sectional width in longitudinal median sections.

### Protein purification and western immunoblots

Carboxysomes were enriched to the Triton-Percoll pellet stage of the Epps-EDTA method for β-carboxysome enrichment as described previously [43], [48]. Crude β-carboxysome preparations were also made using the Mg^2+^ precipitation method [49]. IMAC purification of hexahistidine-tagged protein complexes was carried out as described previously [16] and protein samples were concentrated by precipitation with one volume of 100% trichloroacetic acid on ice for 30 minutes. After incubation the sample was collected by centrifugation at 4°C and washed twice with ice cold 80% acetone. The pellet was finally resuspended in 1x NuPAGE sample buffer and 50 mm dithiothreitol.

Western immunoblots were performed as described previously, using the same CcaA, CcmM-35, CcmK2 (called CcmK1 in our previous work), and RuBisCO antibodies [15]. We have also performed western immunoblots using a polyclonal rabbit CcmO antibody raised against recombinant *S. elongatus* PCC 7942 CcmO. We have previously shown that the RuBisCO antibody reacts to the large and small RuBisCO subunits [16], and that the CcmM-35 antibody reacts with the 35 and 58 kDa isoforms of CcmM [15]. The CcmK2 antibody detects CcmK3 and CcmK4 proteins produced in *E. coli* but not CcmO produced using the same expression system. In contrast, our CcmO antibody specifically detects CcmO without significant detection of CcmK2-4.

## Supporting Information

Figure S1 **Carboxysome ultrastructures in complemented mutant strains.** **A**, the HIND *ccmK2* insertional mutant complemented with pSE41-*ccmK2*. **B**, Δ*ccmK3-4* complemented with pSE41-*ccmK3-4.* **C**, Δ*ccmO* complemented with pH6-Ub-*ccmO*. Scale bars are 500 nm.
(TIFF)

Figure S2 **Photosynthetic O_2_ evolution in response to external Ci by complemented BMC mutants.** Shown are a representative set of mass-spectrometric measurements of Ci-dependent O_2_ evolution by wild type and mutant strains of *S. elongatus* PCC 7942 over a range of Ci concentrations. Ci is the sum of CO_2_ and HCO_3_^−^ in solution (pH 7.9).
(TIFF)

Figure S3 **Carboxysomal proteins in wild-type** ***S. elongatus*** **PCC 7942 and BMC mutants.** Western blots show the presence or absence of carboxysomal proteins in the supernatant (**S**) or carboxysome-enriched pellet (**P**) of clarified cyanobacterial lysates treated with 25 mm MgSO_4_ (Mg^2+^ precipitations).
(TIFF)

Figure S4 **Genetic map of the E. coli/S. elongatus gene expression vector pSE41.** pSE41 contains the pBR322 origin for replication in *E. coli* and the pUH24 origin for replication in *S. elongatus* PCC 7942. pSE41 differs from pSE4 by the insertion of the ampicillin resistance marker in frame with the start codon controlled by the nirA promotor from *S. elongatus* PCC 7942, and by the presence of additional restriction target sites NheI, BmtI, BglII, ScaI and XhoI. Unique restriction sites are shown.
(TIFF)

Table S1 **Putative β-carboxysome genes encoded in β-cyanobacterial genomes.** +, ++ and +++ indicate the presence of one, two and three copies of that homologue respectively. **A**, Hexameric bacterial micro-compartment protein (Pfam00936). **B**, Pentameric bacterial micro-compartment protein (Pfam03319). **C**, Tandem BMC protein. **D**, CcmP is a β-cyanobacterial homologue of CsoS1D, a proposed tandem BMC protein from the α-carboxysome [1], [2].
(DOCX)

Table S2 **Carboxysome ultrastructure and diameter at the widest point in complemented mutant strains.** Diameters are expressed as the mean and standard deviation of a sample size shown here. **A**, carboxysome type is defined as wild-type like carboxysome (**CBX**), and carboxysome-like polar body (**PB**). The number of carboxysome measurements is shown in brackets.
(DOCX)

Table S3 **Mutant strains generated and investigated in this study.**
(DOCX)

Table S4 **DNA oligonucleotides used for PCR and molecular biology.** Restriction endonuclease target sites used for molecular biology are underlined or italicised. M13-f/r were used for DNA sequencing only.
(DOCX)
