# Supplementary figures and images for: Structural Determinants of the Outer Shell of β-Carboxysomes in Synechococcus elongatus PCC 7942: Roles for CcmK2, K3-K4, CcmO, and CcmL
Source: PLoS One. 2012 Aug 22;7(8):e43871. doi: 10.1371/journal.pone.0043871 (PMC3425506; doi:10.1371/journal.pone.0043871)

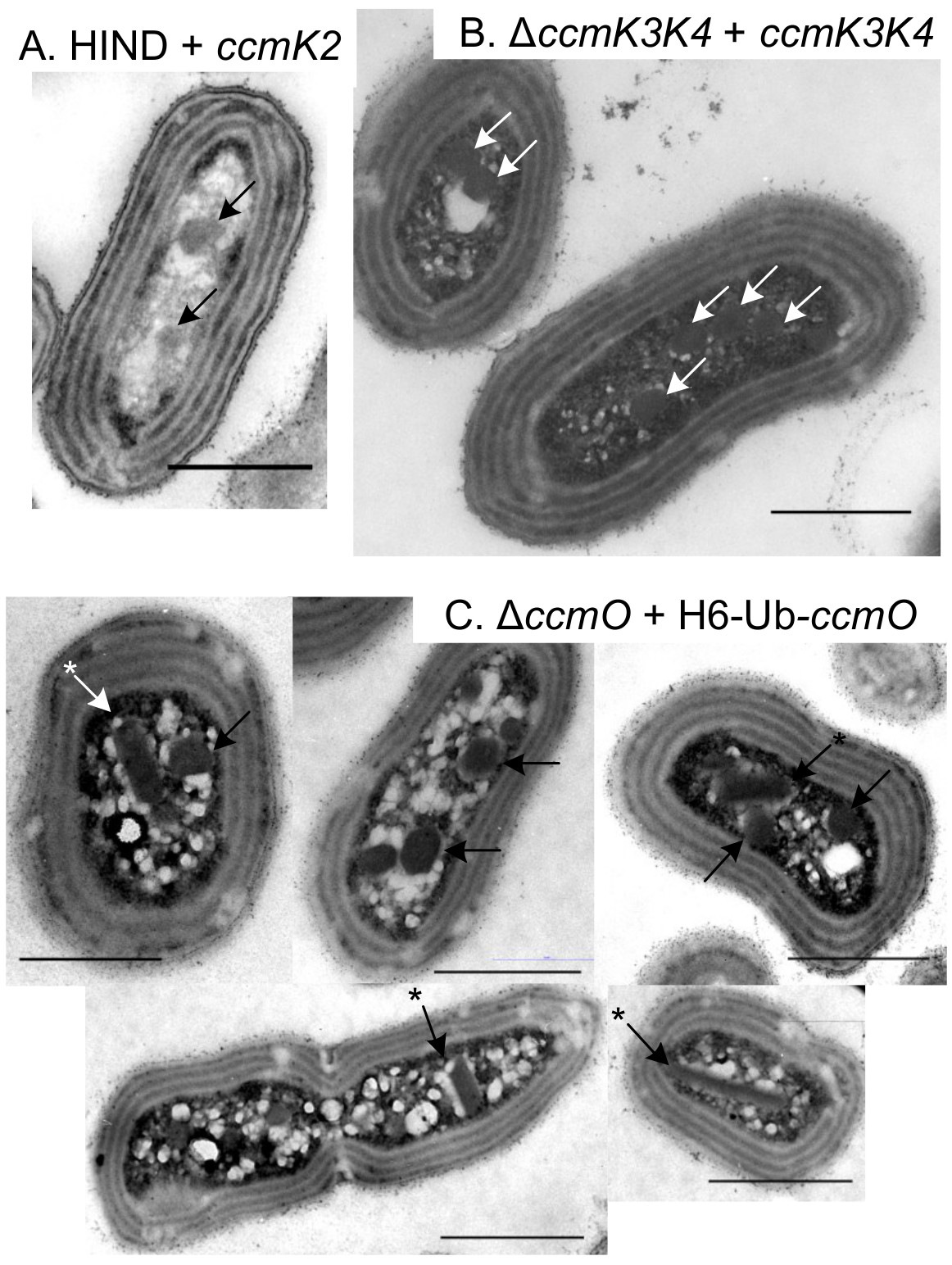

Supplement: Figure S1 — Carboxysome ultrastructures in complemented mutant strains. A, the HIND ccmK2 insertional mutant complemented with pSE41-ccmK2. B, ΔccmK3-4 complemented with pSE41-ccmK3-4. C, ΔccmO complemented with pH6-Ub-ccmO. Scale bars are 500 nm. (TIFF) [file pone.0043871.s001.tiff]

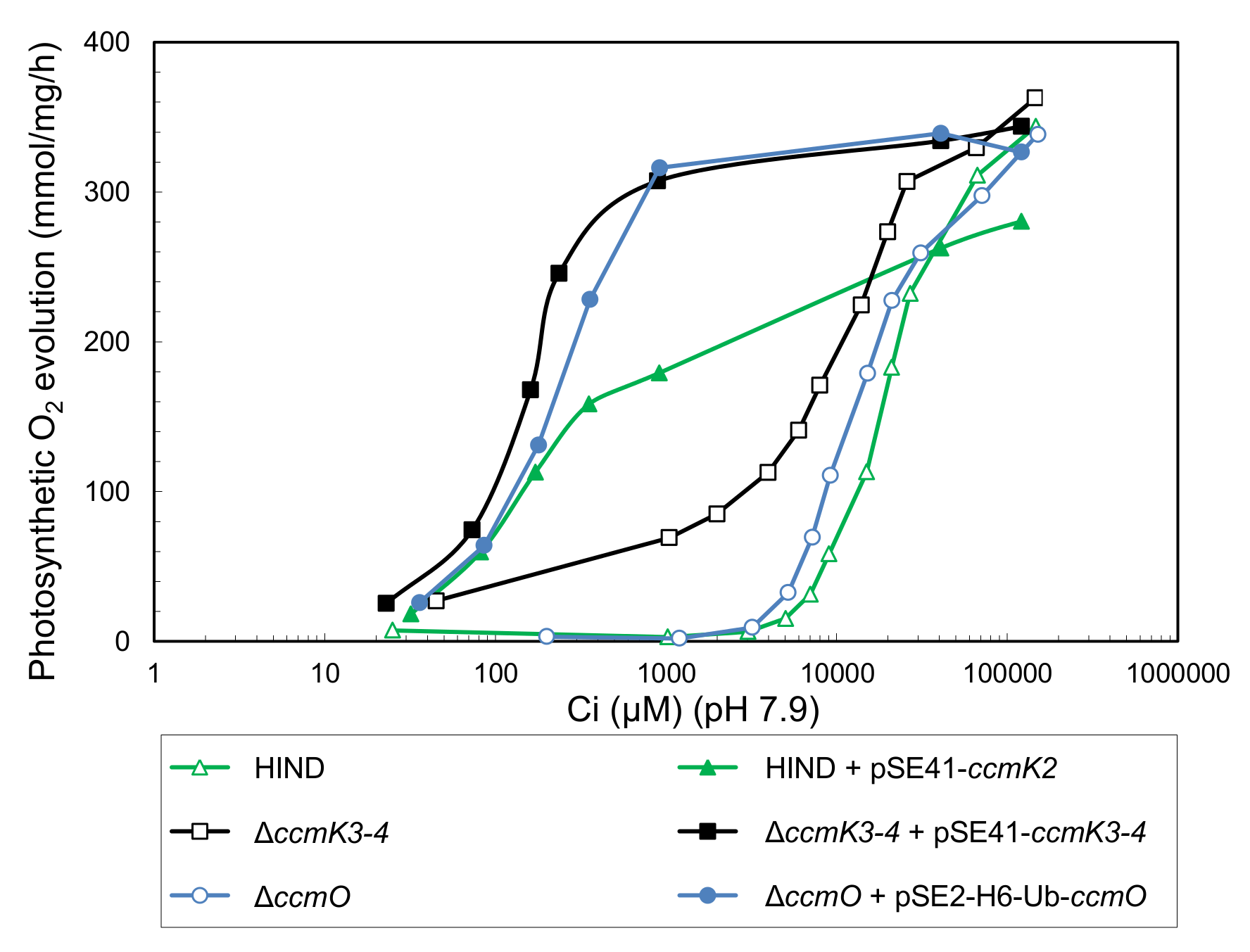

Supplement: Figure S2 — Photosynthetic O2 evolution in response to external Ci by complemented BMC mutants. Shown are a representative set of mass-spectrometric measurements of Ci-dependent O2 evolution by wild type and mutant strains of S. elongatus PCC 7942 over a range of Ci concentrations. Ci is the sum of CO2 and HCO3 − in solution (pH 7.9). (TIFF) [file pone.0043871.s002.tiff]

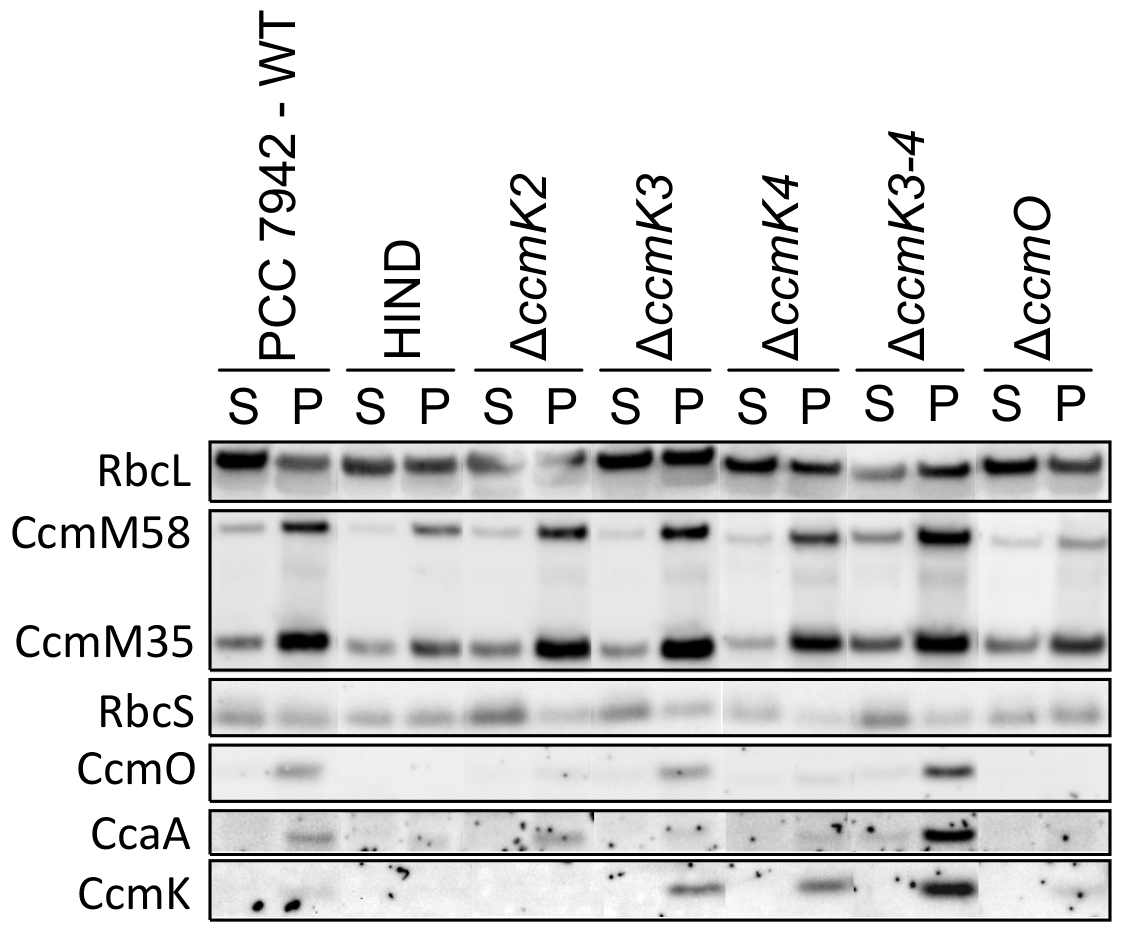

Supplement: Figure S3 — Carboxysomal proteins in wild-type S. elongatus PCC 7942 and BMC mutants. Western blots show the presence or absence of carboxysomal proteins in the supernatant (S) or carboxysome-enriched pellet (P) of clarified cyanobacterial lysates treated with 25 mm MgSO4 (Mg2+ precipitations). (TIFF) [file pone.0043871.s003.tiff]

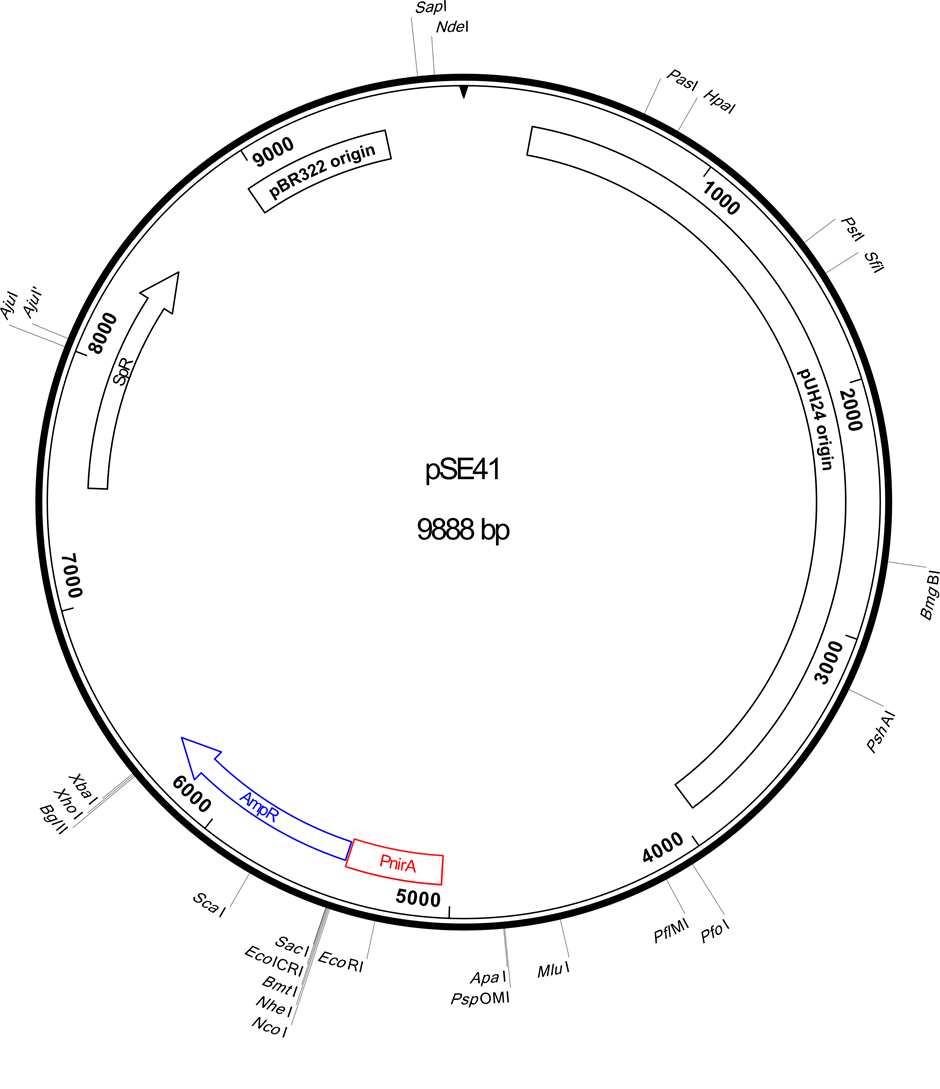

Supplement: Figure S4 — Genetic map of the E. coli/S. elongatus gene expression vector pSE41. pSE41 contains the pBR322 origin for replication in E. coli and the pUH24 origin for replication in S. elongatus PCC 7942. pSE41 differs from pSE4 by the insertion of the ampicillin resistance marker in frame with the start codon controlled by the nirA promotor from S. elongatus PCC 7942, and by the presence of additional restriction target sites NheI, BmtI, BglII, ScaI and XhoI. Unique restriction sites are shown. (TIFF) [file pone.0043871.s004.tiff]
