# Supplementary material for: Structural Determinants of the Outer Shell of β-Carboxysomes in Synechococcus elongatus PCC 7942: Roles for CcmK2, K3-K4, CcmO, and CcmL
Source: PLoS One. 2012 Aug 22;7(8):e43871. doi: 10.1371/journal.pone.0043871 (PMC3425506; doi:10.1371/journal.pone.0043871)
Supplement: Table S1 — Putative β-carboxysome genes encoded in β-cyanobacterial genomes. +, ++ and +++ indicate the presence of one, two and three copies of that homologue respectively. A, Hexameric bacterial micro-compartment protein (Pfam00936). B, Pentameric bacterial micro-compartment protein (Pfam03319). C, Tandem BMC protein. D, CcmP is a β-cyanobacterial homologue of CsoS1D, a proposed tandem BMC protein from the α-carboxysome [1], [2]. (DOCX) [file pone.0043871.s005.docx]

Table S1 **Putative β-carboxysome genes encoded in β-cyanobacterial genomes. +, ++** and **+++** indicate the presence of one, two and three copies of that homologue respectively. **A**, Hexameric bacterial micro-compartment protein (Pfam00936). **B**, Pentameric bacterial micro-compartment protein (Pfam03319). **C**, Tandem BMC protein. **D**, CcmP is a β-cyanobacterial homologue of CsoS1D, a proposed tandem BMC protein from the α-carboxysome [1,2].

| **Strain** | **hBMC^a^** | | | | **pBMC^b^** | **tBMC^c^** | |
| --- | --- | --- | --- | --- | --- | --- | --- |
|  | ***ccmK2*** | ***K1*** | ***K3*** | ***K4*** | ***ccmL*** | ***ccmO*** | ***ccmP^d^*** |
| ***Acaryochloris* *marina* MBIC11017** | + | + | + | +++ | + | + | + |
| ***Anabaena* *variabilis* ATCC 29413** | + | + | + | + | + | + | + |
| ***Arthrospira* *maxima* CS-328** | + | + | + | + | + | + | + |
| ***Arthrospira* *platensis* Paraca** | + | + | + | + | + | + | + |
| ***Arthrospira* *platensis* PCC 8005** | + | + | + | + | + | + | + |
| ***Crocosphaera* *watsonii* WH 8501** | + | + | + | + | + | + | + |
| ***Cyanothece* ATCC 51142** | + | + | + | + | + | + | + |
| ***Cyanothece* CCY 0110** | + | + | + | + | + | + | + |
| ***Cyanothece* PCC 7424** | ++ | + | + | + | + | + | + |
| ***Cyanothece* PCC 7425** | + | + | + | ++ | + | + | + |
| ***Cyanothece* PCC 7822** | ++ | + | + | + | + | + | + |
| ***Cyanothece* PCC 8801** | + | + | + | + | + | + | + |
| ***Cyanothece* PCC 8802** | + | + | + | + | + | + | + |
| ***Cylindrospermopsis* *raciborskii* CS-505** | + | + | + | + | + | + | + |
| ***Gloeobacter* *violaceus* PCC 7421** | + | + |  |  | + | + | + |
| ***Lyngbya* *aesturii* CCY 8106** | + | + | + | + | + | + | + |
| ***Microcoleus* *chthonoplastes* PCC 7420** | + | + | + | + | + | + | + |
| ***Microcystis* *aeruginosa* NIES-843** | + | + | + | + | + | + | + |
| ***Nodularia* *spumigena* CCY9414** | ++ | + | + | + | + | + | + |
| ***Nostoc* *azollae* 0708** | + | + | + | + | + | + | + |
| ***Nostoc* *punctiforme* PCC 73102** | + | + | + | + | + | + | + |
| ***Nostoc* PCC 7120** | + | + | + | + | + | + | + |
| ***Oscillatoria* PCC 6506** | ++ | + |  |  | + | + | + |
| ***Raphidiopsis* *brookii* D9** | + | + | + | + | + | + | + |
| ***Synechococcus* *elongatus* PCC 6301** | + |  | + | + | + | + | + |
| ***Synechococcus* *elongatus* PCC 7942** | + |  | + | + | + | + | + |
| ***Synechococcus* JA-2-3B'a** | + | + |  |  | + | + | + |
| ***Synechococcus* JA-3-3Ab** | + | + |  |  | + | + | + |
| ***Synechococcus* PCC 7002** | + | + | + | + | + | + | + |
| ***Synechococcus* PCC 7335** | + | + | + | + | + | + | + |
| ***Synechocystis* PCC 6803** | + | + | + | + | + | + | + |
| ***Thermosynechococcus* *elongatus* BP-1** | + | + | + | + | + | + | + |
| ***Trichodesmium* *erythraeum* IMS101** | + | + | + | + | + | + | + |

1. Kinney JN, Axen SD, Kerfeld CA (2011) Comparative analysis of carboxysome shell proteins. Photosynth Res 109: 21-32.

2. Roberts EW, Cai F, Kerfeld CA, Cannon GC, Heinhorst S (2012) Isolation and characterization of the Prochlorococcus carboxysome reveal the presence of the novel shell protein CsoS1D. J Bacteriol 194: 787-795.
