# Supplementary material for: Structural Determinants of the Outer Shell of β-Carboxysomes in Synechococcus elongatus PCC 7942: Roles for CcmK2, K3-K4, CcmO, and CcmL
Source: PLoS One. 2012 Aug 22;7(8):e43871. doi: 10.1371/journal.pone.0043871 (PMC3425506; doi:10.1371/journal.pone.0043871)
Supplement: Table S2 — Carboxysome ultrastructure and diameter at the widest point in complemented mutant strains. Diameters are expressed as the mean and standard deviation of a sample size shown here. A, carboxysome type is defined as wild-type like carboxysome (CBX), and carboxysome-like polar body (PB). The number of carboxysome measurements is shown in brackets. (DOCX) [file pone.0043871.s006.docx]

Table S2 **Carboxysome ultrastructure and diameter at the widest point in complemented mutant strains.** Diameters are expressed as the mean and standard deviation of a sample size shown here. **A**, carboxysome type is defined as wild-type like carboxysome (**CBX**), and carboxysome-like polar body (**PB**). The number of carboxysome measurements is shown in brackets.

| **Genotype** | **Carboxysome** | |
| --- | --- | --- |
|  | **Type^A^** | **Diameter (nm)** |
| PCC 7942 | CBX | 175 ± 37 (35) |
| HIND + pSE41-*ccmK2* | CBX | 157 ± 26 (17) |
| Δ*ccmK3-4* + pSE41-*ccmK3-4* | CBX | 147 ± 23 (18) |
| Δ*ccmO* + pSE2-H6-Ub*-ccmO* | CBX | 197 ± 38 (31) |
