# Supplementary material for: Structural Determinants of the Outer Shell of β-Carboxysomes in Synechococcus elongatus PCC 7942: Roles for CcmK2, K3-K4, CcmO, and CcmL
Source: PLoS One. 2012 Aug 22;7(8):e43871. doi: 10.1371/journal.pone.0043871 (PMC3425506; doi:10.1371/journal.pone.0043871)
Supplement: Table S3 — Mutant strains generated and investigated in this study. (DOCX) [file pone.0043871.s007.docx]

Table S3 **Mutant strains generated and investigated in this study.**

| **Genotype** | **Deletion (Locus Tag)** | **Reference** |
| --- | --- | --- |
| ***S. elongatus* PCC 7942 WT** | **n/a** | **[1]** |
| ***ccmK2::Cm^R^*** | Synpcc7942_1421 | **[2]** |
| **Δ*ccmK2::Cm^R^*** | Synpcc7942_1421 |  |
| **Δ*ccmK3::Cm^R^*** | Synpcc7942_0284 |  |
| **Δ*ccmK4::Cm^R^*** | Synpcc7942_0285 |  |
| **Δ*ccmK3-4::Cm^R^*** | Synpcc7942_0284, Synpcc7942_0285 |  |
| **Δ*ccmO::Cm^R^*** | Synpcc7942_1425 |  |
| **Δ*ccmK2::Cm^R^/*pSE4*-ccmK2*** | Synpcc7942_1421 | **[3]** |
| **Δ*ccmK3-4::Cm^R^/*pSE41-*ccmK3-4*** | Synpcc7942_0284, Synpcc7942_0285 | **[3]** |
| **Δ*ccmO::Cm^R^/*pSE2-H6-Ub-*ccmO*** | Synpcc7942_1425 | **[4]** |
| **Δ*ccmM*** | Synpcc7942_1423 | **[5]** |

1. Rippka R, Deruelles J, Waterbury J, Herdman M, Stanier R (1979) Generic assignments, strain histories and properties of pure cultures of cyanobacteria. Microbiology 111: 1-61.

2. Price GD, Howitt SM, Harrison K, Badger MR (1993) Analysis of a genomic DNA region from the cyanobacterium *Synechococcus* sp. strain PCC 7942 involved in carboxysome assembly and function. J Bacteriol 175: 2871-2879.

3. Maeda S, Kawaguchi Y, Ohe T, Omata T (1998) Cis-acting sequences required for *ntcB*-dependent, nitrite-responsive positive regulation of the nitrate assimilation operon in the cyanobacterium *Synechococcus* sp. strain PCC7942. J Bacteriol 180: 4080-4088.

4. Aichi M, Omata T (1997) Involvement of *ntcB*, a *lysR* family transcription factor, in nitrite activation of the nitrate assimilation operon in the cyanobacterium *Synechococcus* sp. strain PCC7942. J Bacteriol 179: 4671-4675.

5. Woodger FJ, Badger MR, Price GD (2005) Sensing of inorganic carbon limitation in *Synechococcus* PCC7942 is correlated with the size of the internal inorganic carbon pool and involves oxygen. Plant Physiol 139: 1959-1969.
