# Supplementary material for: Structural Determinants of the Outer Shell of β-Carboxysomes in Synechococcus elongatus PCC 7942: Roles for CcmK2, K3-K4, CcmO, and CcmL
Source: PLoS One. 2012 Aug 22;7(8):e43871. doi: 10.1371/journal.pone.0043871 (PMC3425506; doi:10.1371/journal.pone.0043871)
Supplement: Table S4 — DNA oligonucleotides used for PCR and molecular biology. Restriction endonuclease target sites used for molecular biology are underlined or italicised. M13-f/r were used for DNA sequencing only. (DOCX) [file pone.0043871.s008.docx]

Table S4 **DNA oligonucleotides used for PCR and molecular biology.** Restriction endonuclease target sites used for molecular biology are underlined or italicised. M13-f/r were used for DNA sequencing only.

| **Primer** | **Construct(s)** | **Sequence** |
| --- | --- | --- |
| **delK2-1f-HindIII** | pUC18Δ*ccmK2* | AAGCTTCTTGTCGAGGGCAAACAAGT |
| **delK2-1r-SacI** | pUC18Δ*ccmK2* | GAGCTCGAGTCCACGTCCTTTAAAAAAGTCG |
| **delK2-2f-SacI** | pUC18Δ*ccmK2* | GAGCTCGGTTGAGGGCTGTTGAGTC |
| **delK2-2r-EcoRI** | pUC18Δ*ccmK2* | GAATTCCTGGCCTATCCAGACGGAAT |
| **delO-1f-HindIII** | pUC18Δ*ccmO* | AAGCTTCAGCCACAGCTACACCTCAA |
| **delO-1r-BamHI** | pUC18Δ*ccmO* | GGATCCAGCTCCTAGAGCTGCTGTGC |
| **delO-2f-BamHI** | pUC18Δ*ccmO* | GGATCCCCTTGGTTTTTGGCTGAAAG |
| **delO-2r-EcoRI** | pUC18Δ*ccmO* | GAATTCTTGGAAGGTTTTGACCAAGG |
| **delK3K4-1f-HindIII** | pUC18Δ*ccmK3,* pUC18Δ*ccmK3-4* | AAGCTTGATAGTCAGGTTGATCCGC |
| **delK3K4-1r-XbaI** | pUC18Δ*ccmK3,* pUC18Δ*ccmK3-4* | TCTAGAGATCGCAGCGGGATGGAAGAG |
| **delK3K4-2f-XbaI** | pUC18Δ*ccmK4,* pUC18Δ*ccmK3-4* | TCTAGAGGGTAGCGACTGATTAAAAGGGTTG |
| **delK3K4-2r-EcoRI** | pUC18Δ*ccmK4,* pUC18Δ*ccmK3-4* | GAATTCTGTCGTCACTGGATAGATGTCACC |
| **delK4-1f--HindIII** | pUC18Δ*ccmK4* | AAGCTTACTGCGGCCATGAAGTGTCAGC |
| **delK4-1r-XbaI** | pUC18Δ*ccmK4* | TCTAGACAGTGTCGATTGTAAAGTCC |
| **delK3-2f-XbaI** | pUC18Δ*ccmK3* | TCTAGAGCCACGGACTTTACAA |
| **delK3-2r-EcoRI** | pUC18Δ*ccmK3* | GAATTCCAAAAGCCAATTGGTCATCGT |
| **K2-exp-f** | pSE41-*ccmK2* | ACCACGCTTGGTTGAGAAAC |
| **K2-exp-r** | pSE41-*ccmK2* | AACGACTCAACAGCCCTCAA |
| **K3K4-exp-f** | pSE41-*ccmK3-4* | TCAGAGAACCTACCTGCAGT |
| **K3K4-exp-r** | pSE41-*ccmK3-4* | TAGGTCCGTTTTCCAACAGG |
| **O-exp-f-BamHI** | pSE2-H6-Ub-*ccmO* | GGTACTGGATCCATGTCGGCTTCTCTTCC |
| **O-exp-r-XbaI/HindIII** | pSE2-H6-Ub-*ccmO* | TTAAGCT*TCTAGA*TTACTGATCATCACGAGGATTGG |
| **M13-f** |  | GTTTTCCCAGTCACGAC |
| **M13-r** |  | CAGGAAACAGCTATGAC |
